# Co‐Creation Methodology for Developing a Racial Inclusivity Training Resource in Physiotherapy Education

**DOI:** 10.1111/hex.70363

**Published:** 2025-08-04

**Authors:** Yetunde Dairo, Meriel Norris, Annabel Williams, John A. Hammond

**Affiliations:** ^1^ School of Health and Social Care Professions Buckinghamshire New University High Wycombe UK; ^2^ College of Health, Medicine and Life Sciences, Department of Health Sciences, Mary Seacole Building Brunel University London Uxbridge UK; ^3^ School of Sport, Nutrition and Allied Health Professions Oxford Brookes University Oxford UK; ^4^ Centre for Allied Health St George's University of London London UK

**Keywords:** co‐creation, design thinking, physiotherapy education, racial inclusivity, training resource

## Abstract

**Background:**

Attainment disparities and experiences of racism among physiotherapy students from racially minoritised groups highlight the need for inclusive training resources. Co‐creation actively involves end users in the development process, ensures relevance and efficacy, and was considered necessary for creating such a resource.

**Objectives:**

This paper outlines co‐creation methodology and processes in developing a training resource to raise awareness of racial discrimination and challenge practice in physiotherapy practice education. It examines the potentialities and challenges of co‐creation within this context.

**Participants:**

Physiotherapy students (*n* = 5), practice educators (*n* = 5) and project team members (*n* = 4) participated, ensuring diverse representation.

**Methods:**

A design thinking approach guided the process, beginning with an initial scoping exercise with stakeholders (*n* = 60), followed by six online workshops. Workshops focused on defining key issues, integrating insights from prior interviews, and developing prototypes. The final stages involved iterative refinement following pilot testing of the resource.

**Main Findings:**

The co‐creation process was underpinned by principles of inclusivity, collaboration, support and safety. It involved exploration, scope‐setting, delivery and evaluation, reflecting the commitment of all parties.

**Discussion:**

Key considerations include resource sustainability, time investment and challenges in quantifying co‐creation's impact. The project highlights the ongoing role of co‐creators in maintaining resource relevance and promoting systemic change.

**Conclusion:**

This paper provides a review of a methodology for co‐creating a racial inclusivity training resource in physiotherapy education. The approach demonstrates its potential, offering a valuable reference for future inclusivity initiatives.

**Public Contribution:**

Co‐creators, including physiotherapy students and practice educators from racially minoritised backgrounds, contributed their lived experiences to all stages, shaping the resource to reflect real‐world challenges and solutions. Their involvement ensured authenticity and impact.

## 1 Introduction

Co‐creating curricula by students and staff has been recognised as a crucial approach that fosters meaningful learner engagement and facilitates positive student–staff relationships [1]. Co‐creation is defined as a process where students and staff collaboratively contribute to curriculum development, resulting in educational experiences that are responsive to diverse needs [2, 3]. These approaches emphasise shared decision‐making, collective wisdom and diverse perspectives of all stakeholders, aiming to create inclusive and responsive educational experiences. Guachalla and Gledhill [4] argue that different contributors bring their unique perspectives, shaped by their understanding of individual potential, academic prerequisites, regulatory guidelines and current market demands. These diverse yet complementary perspectives are likely the reason why co‐creation is a valued approach for pedagogic design.

Co‐creation is not only a process but can also be a methodology. As a process, it aims to collaboratively generate outputs [5], while as a methodology, it systematically generates knowledge and insights through ongoing collaboration [6]. Research has shown that co‐creating curricula has a transformative impact on education [1, 4] and an equally profound impact on individuals and communities [7, 8, 9]. For instance, Corcoran et al. [9] found that co‐creation workshop activities supported psychological and community well‐being changes by enhancing both personal growth and a collective sense of optimism in participants. However, co‐creation also presents several challenges, including balancing diverse perspectives, overcoming power differentials, ensuring diverse representation [10, 11] and managing varying levels of expertise [4]. These challenges highlight the importance of inclusivity, both in the outcomes and in the processes of co‐creation [5, 6]. When addressing a project focused on racial inclusivity, these co‐creation principles are both appealing and essential, yet they also present specific complexities that must be navigated.

The under‐representation of minority ethnic groups in research projects in the United Kingdom has been the focus of several studies and reports, with various barriers identified as hindrances to their participation. Some of these barriers specific to this project are: historical abuses and exploitation in research, limited outreach and communication efforts targeted at minority communities, under‐representation of minoritised groups as researchers, practical issues such as financial constraints and transportation difficulties, and a general mistrust in healthcare and academic institutions conducting research [10, 12, 13, 14, 15]. While the co‐creation methodology reported in this paper is about educational development, we discuss efforts to address some of the issues identified in research co‐creation, specifically in terms of targeted communication, ensuring representativeness of co‐creators and tackling practical issues such as financial constraints and transportation difficulties. Moreover, the presence of distrust in healthcare and academic institutions conducting projects with concerns over data privacy and confidentiality is recognised and addressed.

### 1.1 Context of Physiotherapy Education

In the United Kingdom, physiotherapy education is usually a 3‐year BSc (Honours) or a 2‐year MSc for those with an undergraduate degree in related fields like sports, healthcare or science. Recognised by the Health and Care Professions Council, these programmes allow graduates to register immediately. Students undertake significant practice placements (1000 h) in clinical settings, supervised and assessed by trained physiotherapists who have completed specific training provided usually by a university.

The field of physiotherapy education is evolving. In the United Kingdom, there is an increasing number of students from racially minoritised backgrounds studying physiotherapy. In 2021/22, when the project commenced, figures demonstrate 15.5% of UK domicile students are from racially minoritised backgrounds [16], which is beginning to reflect proportions (17.8%) in the Census in England and Wales [17]. However, university and practice educators remain predominantly white due to historical and professional cultural systems that have privileged whiteness. In these systems, whiteness has been viewed as the ‘norm’, against which other racial identities are defined [18]. Research in physiotherapy education has demonstrated that, in comparison to white students, those who are racially minoritised are likely to be awarded lower marks in observed assessments, including placements and have lower odds of being awarded a good degree classification [19]. To contextualise this further, physiotherapy students from racially minoritised backgrounds report experiences of racism and a struggle with a sense of belonging during their courses, including placements [20]. To effectively address these challenges, the participants in Hammond et al. [20] asserted that practice educators require more effective training on racial inclusivity, and they wanted their voices and experiences to be at the centre of any intervention.

To address these challenges, funding was secured from Health Education England in 2021 to develop a training resource aimed at promoting racial inclusivity in practice education [21]. The resource aimed to support practice educators in tailoring supervision methods to be culturally responsive and sensitive to the diverse needs of their student population. Apart from the desire to incorporate the lived experiences of racially minoritised students, the format and content of the resource were deliberately not developed to enable a nuanced and context‐specific co‐creation process with the active participation of multiple stakeholders, including academics, practice placement educators and physiotherapy students [22].

### 1.2 What This Paper Will Do

This paper follows the development of the racial inclusivity training resource, which can be accessed via Hammond et al. [21] and an evaluation report of the pilot implementation by Dairo et al. [22]. Therefore, this sets the stage for the current paper, which provides a detailed exploration of the co‐creation methodology used in developing the racial inclusivity training resource within physiotherapy education. It aims to clarify the process, reflections and outcomes of co‐creating an educational resource on a sensitive topic, race and discrimination, where the broader context of healthcare education and the potential power differentials of members of the co‐creator group were key considerations. In this paper, co‐creation is a collaborative and participatory process that actively engages physiotherapy academics, practice placement educators and physiotherapy students in developing an educational resource. A subsequent paper will discuss the longer‐term impact of the methodology on the co‐creator participants.

The next section will explore the intricacies of the methodology, its components, the challenges encountered and its transformative potential for fostering racial inclusivity in physiotherapy education. We will discuss how the structured interactions between physiotherapy academics, practice placement educators and students created a platform for dialogue, shared decision‐making and mutual learning. Through this exploration, we aim to contribute to the broader discourse on collaborative methodologies for promoting inclusivity in healthcare education.

## 2 Methodology and Methods

### 2.1 Theoretical Underpinnings

Several methodologies are commonly employed in co‐creation research, including Stakeholder Advisory Panels [23], Participatory Action Research [24, 25] and Co‐Design Workshops [26, 27]. Our project employed the Co‐Design Workshops, whose methodology aligns with established co‐production principles, particularly those articulated by Norström et al. [28], ITHS [29] and NHS England [30], that emphasise salience, credibility, legitimacy and inclusivity. These principles provided a theoretical framework for structuring the co‐creation process, ensuring that the resource developed was not only relevant and practical but also equitable, with transparency in its process and had resonance for those with lived experience. This alignment underscores the value of integrating diverse perspectives and fostering mutual learning throughout the research process.

### 2.2 Design

A design thinking approach informed the co‐creation methodology used in this project, a problem‐solving framework prioritising human‐centric, innovative and collaborative processes [7, 26, 31]. When applied to co‐creation, design thinking emphasises collaboration among diverse stakeholders to address complex challenges and create solutions that deeply resonate with end users. It is commonly used in educational research involving co‐creation [32, 33] as it emphasises collaboration, creativity and iterative problem‐solving, aligning stakeholders' efforts towards addressing challenges in a human‐centred and inclusive manner.

To commence the co‐creation methodology in this project, we held an initial scoping meeting with a range of stakeholders (*n* = 60) who were known experts or allies in healthcare and social justice to communicate the project's intentions and seek feedback on the co‐creation approach. The stakeholders reiterated the need to address the specific imbalance of white privilege and power in the development group. They also strongly supported the intention to co‐create and emphasised the importance of appropriate representation from minoritised groups, the meaningfulness of contributions, and fair remuneration. A transparent recruitment process for the co‐creators followed this.

The actual resource development process involved several phases over 9 months. The co‐creators were involved in all phases of the project, from the development of the inclusivity resource training tool through to the finalisation of the inclusivity resource. The co‐creators identified and supported the logistics of the pilot testing, and the evaluation was conducted by one of the co‐creators, who was employed as a research assistant.

### 2.3 Participants

The participants in this co‐creation initiative were purposefully selected to ensure representation from diverse perspectives and to ensure their availability during the project duration. There were physiotherapy students from racially minoritised backgrounds (*n* = 5), practice educators also from racially minoritised backgrounds (*n* = 5) and academic project team members (*n* = 4), three who identify as white, and one from a racially minoritised background. Together, they formed a cohort to collaborate on developing a resource for racial inclusivity training.

### 2.4 Recruitment and Advertising

The recruitment process targeted physiotherapy students from racially minoritised backgrounds and practice educators. An advertisement (see Supporting Information) was placed on the UK Chartered Society of Physiotherapy website, outlining the project's objectives and expected commitments to potential co‐creators. The key expectations contained in the advert are listed below:

- Participants are expected to contribute through co‐design activities, attend up to seven scheduled online meetings, and assist in developing, implementing and evaluating the resource.
- Eligible candidates include qualified UK physiotherapists with practice education experience and pre‐registration students from racially minoritised backgrounds with prior placement experience.
- The project involves collaboration within a structured timeline and includes financial compensation for participants.

### 2.5 Eligibility and Selection

Individuals were invited to submit an expression of interest, which was reviewed by the research team. We received a total of 20 expressions of interest, of which 9 were physiotherapy students, 9 were practice educators, and 2 were physiotherapy academics. Co‐creators were selected based on their alignment with the project's objectives and their willingness to actively participate in all three phases. The inclusion of physiotherapy students from racially minoritised backgrounds was to ensure peer views and a diversity of perspectives in the co‐creation process.

### 2.6 Conditions of Engagement

Following the agreed commitment to join the co‐creator group, contractual arrangements were set up to enable students and practice educators to be paid for their time. The students were employed and compensated as research assistants, while the educators were employed and compensated as research fellows, in accordance with the host organisation's practices. Guidance on roles, responsibilities and expected time commitments was communicated in advance of the first meeting.

An early consideration was the power differentials between the existing academic team, who had secured the funding, and the newly recruited co‐creators. The academic colleagues met regularly in advance of the first meeting with all co‐creators to reflect on and plan the principles of collaboration to facilitate a welcoming, non‐judgemental and inclusive environment.

- The first meeting collaboratively created ground rules for communication and engagement, which were agreed.
- The project team ensured that all participants were treated as equals, regardless of their background, and that the atmosphere was welcoming and non‐judgmental.
- All meetings were chaired by one of the academic project team members.
- The agenda was semi‐structured.
- Co‐creators were offered a debriefing opportunity by one of the project team at the end of each meeting.
- To ensure that all relevant voices were heard, alternative means of communication other than verbal were agreed. For example, co‐creators were given the option to send responses by email either before or after each online meeting.
- There was a review meeting after all the workshops had concluded.

### 2.7 Development of the Racial Inclusivity Resource Training Tool

**Figure 1:**
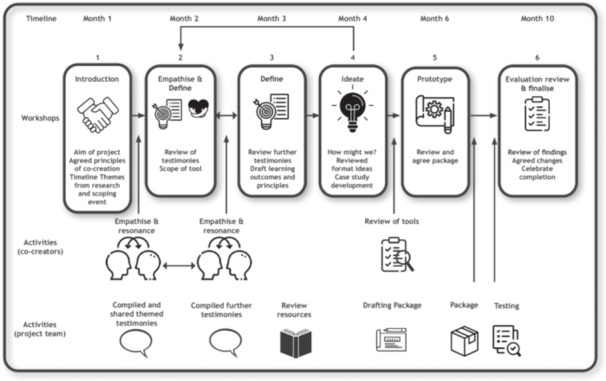
Representation of workshop phases and participant activities.

The tool was developed with co‐creators through a series of workshops, comprising six sessions spread across 9 months, beginning in November 2021. The workshops were held approximately every 4 weeks, with a longer gap for evaluation between workshops 4 and 5. A final workshop was held following the evaluation of the tool to review results and finalise. The design thinking steps used in each workshop are illustrated in Figure 1. Due to the global COVID‐19 pandemic and for accessibility, workshops were conducted online, leveraging video conferencing as the primary mode of communication. This method was an acceptable alternative to face‐to‐face groups and allowed people from various geographical locations to attend. The workshops lasted 1–1.5 h, and one‐to‐one or small group catch‐up sessions were facilitated for those unable to attend. Communication by email and/or telephone was also available if needed.

### 2.8 Introduction and Setting the Scene (Workshop 1)

Workshop 1 focused on establishing ground rules and initiating brainstorming to identify key issues related to racial discrimination and inclusivity. The objectives were to create a welcoming environment for co‐creators, clarify the intended outcomes of the project, help contributors understand the design process and their roles within it, discuss how they could contribute, collaborate to draft an agreement for cooperation, and plan the next steps to ensure a successful outcome. A range of activities, such as mapping current knowledge through research and the initial scoping event, alongside the collaborative development of shared group behaviours, were employed to engage and facilitate participant interaction. Furthermore, the explicit acknowledgement of positionality and the sensitivity of the subject matter were discussed. This was particularly relevant as a key direction from the research involved using real student testimonies in the educational tool. While an academic co‐creator chaired the workshop, a conscious effort was made to provide concise verbal commentary to amplify the voices of other co‐creators. A summary of the meeting was prepared and shared via email with all co‐creators, inviting them to suggest changes or additions.

### 2.9 Empathise and Define (Workshop 2)

Workshop 2 focused on the Empathise and Define stages. Before the session, co‐creators accessed anonymised student testimonies about practice placements [34]. The students, from racially minoritised backgrounds in various UK healthcare settings, had given full consent (Norris et al. 2019). These insights helped co‐creators understand student experiences, challenges and needs. It was also noted that, while testimonies formed the basis for the training resource, its structure and format were still to be determined, allowing participants to exercise creativity and ownership.

During this workshop, co‐creators considered these verbatim quotes, selected, and collectively prioritised those that both resonated and covered a broad range of experiences. The quotes identified several microaggressions, including ‘microassaults’ (discriminatory verbal abuse), ‘microinsults’ (insensitive and disparaging comments) and ‘microinvalidations’ (dismissive and exclusionary practices) [35]. They included quotes from both male and female students who had experienced microaggressions from patients, as well as their reflections on how the practice educator managed these situations. Other quotes reflected student accounts of bias in assessment, with students from racially minoritised backgrounds describing a negative impact on attainment in the practice setting compared to their white peers. A sense of ‘otherness’ was captured in several quotes, affecting students' sense of belonging within the physiotherapy profession, alongside reports of racial and cultural stereotyping, internalised expectations, and increased personal and professional labour within the workplace.

The co‐creators noted that there were limited examples of positive experiences and assumptions regarding language capability, which they believed were relevant based on their own experiences. It was agreed that the original transcripts would be reviewed again for potential examples. A summary email was sent following the workshop, which also included signposting for care, given the sensitive nature of the topic discussed.

### 2.10 Define (Workshop 3)

While the initial plan for this workshop was to proceed to ideation, a need arose to refine the focus of the educational tool further. Before the workshop, co‐creators received additional testimonies, which were collectively reviewed and prioritised. The overarching themes of the testimonies were agreed upon, which would inform the main activities within the tool.

Discussions regarding the overall learning outcomes of the educational tool developed, alongside broader structural concepts, such as the necessity for the educational tool to be viewed as part of the learning journey. The mapping of the overall programme flow was initiated.

### 2.11 Ideate (Workshop 4)

In Workshop 4, we moved to the Ideate stage, where participants rethought the educational tool's goals and issues from Workshop 1, turning them into actionable ‘how might we’ (HMW) questions. These questions guided co‐creators in developing a conceptual framework, including orientation information, case studies and discussions. Space for reflection and a ‘what next’ was included for ongoing development. Resources from other training packages were shared as templates, along with a provisional format.

Following Workshop 4, the academic team drafted a prototype education tool. This included arranging for filming of the selected testimonies, drafting pre‐session activities, providing post‐session guidance, and selecting the case studies to be used. Specific tools, including a selection of videos and case studies, were shared with the co‐creators before the next workshop.

### 2.12 Prototype (Workshop 5)

Workshop 5 reviewed the prototype, which included an electronic mock‐up and content such as student testimonies and case studies. Feedback led to the addition of subtitles and revisions to the wording. Suggestions to improve pre‐session info with short videos on concepts like micro‐aggression were incorporated. A final visual map was approved. The academic team then completed the full educational tool—facilitator guides, slides and materials—based on workshop feedback. They planned the evaluation, obtained ethics approval, recruited sites, conducted pilot trainings and assessed impact. Details are in Dairo et al.'s publications [11, 22].

### 2.13 Evaluation, Review and Finalise (Workshop 6)

Workshop 6 was held approximately 4 months later and focused explicitly on reviewing the final resource in light of the evaluation report [22] from the two pilot sites. The full education tool was shared in advance, along with the findings of the evaluation. Responses regarding refinements were discussed, and solutions were agreed upon. Following the workshop, the project team undertook further refinements to finalise the resource.

After the last workshop, the project team held a face‐to‐face gathering to celebrate the co‐creators' contributions. Through small groups and post‐it notes, co‐creators shared their experiences and ideas for sharing the resource. Questions arose about dissemination and co‐creators' future involvement after the project ends, prompting further dialogue.

## 3 Results

There were six workshops lasting a total of 7 h. The concepts underpinning each workshop and the methodologies are summarised in Table 1. The co‐creators' attendance was consistent, except on one occasion when two co‐creators were unable to attend but provided written feedback. All contributions adhered to the time commitment agreed upon in advance. As a result, a training resource was created to support an interactive session for practice educators. It provides a training package to help educators understand the current context, reflect on the experiences of minority students, and consider inclusive responses [21].

**Table 1:** Summary of concepts supporting each workshop, aligned with design thinking principles.

Pre‐workshop activities (before workshop 1)	Workshops 1 and 2: Understand the audience, context and needs	Workshop 3: Define and focus	Workshop 4: Develop prototypes	Workshop 5: Test and refine solutions (part 1)	Workshop 6 Test and Refine Solutions (Part 2)
Stakeholder engagement to shape project intentions and gather feedback Transparent recruitment process for co‐creators Clarification of expectations, roles and contractual arrangements Early planning to promote inclusive engagement	Establishing ground rules for collaboration Exploring power, privilege and positionality Reviewing and discussing student testimonies Identifying key issues (e.g., microaggressions, bias, stereotyping and belonging) Prioritising experiences for inclusion in the resource	Educational tool focus Brainstorming responses to identified challenges Shaping early ideas for content, structure and learning outcomes	Developing ‘How Might We’ (HMW) questions based on selected themes	Reviewing and critiquing the prototype resource Refining content, language and accessibility Enhancing pre‐session orientation materials (e.g., conceptual videos) Confirming structure and user experience	Reviewing evaluation findings from pilot training Agreeing on refinements to finalise the tool Discussing sustainability and future use Planning dissemination and ongoing co‐creator involvement
			Drafting initial components of the resource (e.g., case studies, videos and reflection prompts) Structuring the learning journey		

The main findings emphasised the principles embraced by the co‐creators: inclusivity, collaboration, support, acknowledgement and safety. This was illustrated by the iterative feedback received from the co‐creators throughout the project. As an early example, at the end of Workshop 2, several participants noted the lack of representation of positive experiences in the narratives presented and the absence of examples where language competence was a key factor. Acknowledging this, the project team reviewed the transcripts once more. This resulted in new excerpts being shared with the co‐creators ahead of workshop 3, along with an adjustment to our schedule to allow for an additional round of ‘empathise and resonance’.

Similarly, during workshop 2, it became evident that engaging with the student transcripts had re‐ignited old and challenging memories for many members of the co‐creator group. This was openly discussed and shared during the meeting. Recognising this, a member of the academic team offered individual debrief sessions as needed to acknowledge individual needs and create a safe space for discussing the challenges of the subject matter on a personal level.

By workshop 4, anecdotal experiences became central to the ideation process, guiding decisions on priority narratives and providing space for reflection and self‐compassion. Using ‘HMW’ questions, the project advanced into tool development. Co‐creators drew on their training and safety concerns, emphasising that the training should be a journey of personal and collective growth, not a one‐off. Consequently, a pre‐reading section with conceptual videos was added for potential trainees. Case studies were also updated to focus on ‘what can I do’ discussions, supporting the practical application of strategy.

The co‐creation process involved identifying the needs of all participants, defining the scope of the project, developing a training resource, and jointly evaluating the effectiveness of these resources. This approach attempted to ensure that everyone's needs were considered, project goals were clearly defined, and the impact of the training resources assessed collaboratively.

Specific outcomes developed within the co‐creation process were explored and contextualised in a final face‐to‐face meeting with all the co‐creators involved in this project. For the students and practice educator participants, the discussion of ‘feelings of being involved’ in the project (reflecting the empathise and define phase of the project) reflected the personal and professional experiences of the co‐creators, primarily the importance of lived experience, which was shared in a safe space, even when working online. One participant benefited from working within a home environment, as this provided a point of quiet reflection after the workshops. It was anticipated that there may have been some tensions or contrary views that may not have been expressed earlier in the co‐creation process due to power differentials. However, in the final face‐to‐face session, no tensions or challenges were noted.

For the academic staff, there were shared positive experiences in working with the co‐creators and pleasure in developing actions in this study based on lived experiences of education. The academic staff described the benefits of multi‐institutional collaboration, working with a diverse group in developing a resource for the physiotherapy profession. Academic staff expressed trust and confidence in the development of the educational tool, both in the process underlying its development and in the educational resource itself. Despite this, it was noted that the activities that occurred between sessions (see Figure 1), specifically drafting and finalising the training package and the evaluation, required a significant time commitment from the academic team, which was anticipated; however, the volume was more substantial. While great care had been taken to ensure the time requested of the co‐creators was covered both in contractual terms and remuneration, this was not the case for the academic team. Therefore, additional work outside the standard workshops was absorbed by the academic team, as it was perceived unfair to expect the co‐creators to take on additional work pro bono.

## 4 Discussion

### 4.1 The Co‐Creation Methodology: A Bridge Between Theory and Practice

Co‐creation methodologies, particularly Co‐Design Workshops, have been widely applied in educational research, with varying levels of success and challenges. Existing literature highlights the potential of co‐creation to enhance stakeholder engagement, foster a sense of ownership and improve the relevance of research outputs [26, 27]. However, scholars have also acknowledged the complexities inherent in these approaches, such as power imbalances, tokenism and the sustainability of co‐created resources [9, 24]. Our project aligns with these discussions while offering a distinctive perspective on co‐creation application in physiotherapy education, particularly in addressing racial inclusivity.

By intentionally fostering collaboration, this project navigates the delicate interplay between theory and implementing co‐creation. Physiotherapy education, with its structured hierarchies and distinct challenges, particularly for students from minoritised backgrounds, provides a rich context for exploring the efficacy of co‐creation. The adaptability of co‐creation to developing educational resources in this field underscores its relevance, but our findings highlight some of the limitations that require further consideration.

### 4.2 Collaborative Efforts: Nurturing Inclusivity and Addressing Diverse Learning Needs

Our findings align with previous research indicating that co‐creation enhances students' agency, confidence and ability to contribute authentically by reducing hierarchical dynamics [23, 36, 37, 38]. However, studies have also identified structural barriers that may impede genuinely equitable collaboration [24] and limit decision‐making authority in co‐creation processes, increasing the risks of tokenism and role ambiguity [39, 40]. The essence of co‐creation lies in collaborative efforts among educators, students and academics. Together, they shape pedagogical approaches, content and assessment methods. In our context of physiotherapy practice education, this collaboration becomes an essential investment. By actively engaging with students, we aimed to dismantle systemic barriers that perpetuate racism and microaggressions during practice placements. The resulting co‐created resource reflects the multifaceted nature of contemporary healthcare education. It inherently embeds inclusivity, ensuring that the learning experience resonates with the diverse backgrounds and aspirations of minoritised student groups. In our project, we endeavoured to balance power dynamics and ensure that students' contributions were recognised beyond tokenistic participation. The students actively shaped the resource and its evaluation, but we acknowledge that a sustained formal commitment is still needed from educational institutions or healthcare organisations to integrate these contributions into curricula and policies. Without ongoing efforts, co‐creation risks becoming a temporary intervention rather than a transformative practice [9].

### 4.3 Learning From the Co‐Creation Process

The co‐creation process provided specific learning, and our project highlights the importance of triangulating student, practice educator and academic staff perspectives. While co‐creation projects often emphasise student engagement, our findings reinforce the significance of interdisciplinary input in achieving meaningful outcomes [26]. Academic staff provided multiple perspectives, enriching the co‐creation experience; however, our findings also revealed a disparity in time commitment and engagement levels. Similar to previous studies [25], our work suggests that academic and professional contributors often bear a disproportionate workload, which can lead to frustration and impact the sustainability of co‐creation initiatives. In this project, academic team members worked closely to negotiate and provide technical and visual feedback on specialist video clips created to reflect verbatim student experiences. The exploration and development of these videos took several additional hours. Participatory work specifically recognises the expression of reflectivity, and significant time was committed by both academic collaborators and members of the co‐creation group in reflecting on their own and others' lived experiences of racism [41].

The successful development of the co‐created resource required consideration of sustainability issues, specifically how to ensure the long‐term relevance of the resource. This remains a widely recognised challenge in the literature [9]. While our project successfully engaged participants in developing the training resource, questions remain about how to maintain and update it over time. Co‐creators' involvement was time‐limited due to finite funding resources, and without clear institutional mechanisms for ongoing engagement, there is a risk that the resource will lose relevance. Our findings align with previous studies that have advocated for structural changes to integrate co‐created resources into formal educational frameworks [23].

In balancing the diverse perspectives in this co‐creation, navigating challenges was a key consideration. Time commitment emerged as a key challenge in the project, reflecting broader concerns in co‐creation research about workload distribution and equity [24]. Academic staff invested significantly more time than other co‐creators, approximately five times greater than the others, partly because it aligned with their work responsibilities and values and also due to their accountability to the funder. While this emphasises the benefits of establishing and recruiting co‐creators in the proposal and grant application so that they also possess a sense of ownership, there remains tension since this study is likely to be unfunded, and for students, it is likely to be extracurricular, while for practice educators, these are not directly part of their roles and responsibilities. This raises questions about the feasibility of scaling such initiatives. While our work recognises imbalance in the time committed between the academic and the student and practice educator co‐creators, we have not fully evaluated or understood the emotional labour involved. Indeed, evidence suggests that minoritised students shoulder significant burdens in advancing co‐creative initiatives, leading to fatigue and frustration if institutional follow‐through is weak [15, 41].

This co‐production specifically sought the lived experience of qualified and student physiotherapists from racially minoritised backgrounds, and there was initial recognition of the potential for power differentials between the existing academic staff and the newly formed co‐creator group. Overcoming this and ensuring diverse representation in this project was a key focus, successfully achieved through the development and agreement of conditions of engagement. The success of the co‐production was reflected in the effective communication that underpinned the development and subsequent implementation of the resource. Additionally, although the project has ended, the co‐creator group continues to communicate and provide a resource for guidance and support. While the involvement of racially minoritised students is central to addressing specific issues of racial inclusivity, we recognise that excluding non‐racially minoritised educators from the co‐creation process limits the applicability and representativeness of the resource. These educators are key stakeholders who will benefit from training and help in creating inclusive environments. Their insights into the necessary knowledge and support for fostering inclusivity are crucial. Excluding them risks reinforcing a divide between those needing inclusion and those needing education. A more inclusive approach, one that engages all educators, can promote shared responsibility and collective engagement in advancing racial inclusivity.

Another key point was that our process deviated from the traditional linear co‐design model, instead requiring iterative refinement cycles. While beneficial in ensuring resource fidelity, this non‐linearity poses logistical challenges that require careful planning [25].

### 4.4 Post‐Workshop Insights: Learning From Co‐Creators

Consistent with existing literature, our student and practice educator co‐creators reported a short‐term sense of belonging and empowerment [23, 42, 43, 44]. However, research suggests that these benefits may not be sustained without a formalised commitment to follow‐through [9]. Future studies should explore the long‐term impact of co‐creation on students and practitioners, particularly through longitudinal research examining sustained engagement and systemic change. Our findings contribute to this discussion to emphasise the need for strategic investment in co‐creation methodologies to ensure their lasting impact on education and practice. A forthcoming paper will explore the long‐term impact of the co‐creation process for all participants.

As we look to the future, maintaining the resource's relevance is crucial. Therefore, we are exploring various approaches to communicate and facilitate implementation, and we are interested in how the resource can be applied in other disciplines and contexts. In addition, we are investigating healthcare education to identify approaches that move beyond awareness raising to upskilling practitioners and students to address inequities. Of course, there is a need to consider intersectionalities and other minoritised groups, and we encourage subsequent studies to expand on our findings, contributing to a more just and knowledgeable physiotherapy education sphere.

## 5 Conclusion

Our methodological paper has detailed the co‐creation of a training resource to combat racial discrimination and promote inclusivity in physiotherapy education. Through a multi‐stage methodology, we engaged racially minoritised physiotherapy students and practice educators as co‐creators, fostering a collaborative environment from start to finish. Our recruitment strategy effectively assembled a diverse group, while the development and pilot testing phases saw active participation, leading to a resonant and relevant training resource. The evaluation methods provided comprehensive feedback, enhancing our understanding and acknowledging the significant time investment by all involved.

The implications of our work extend beyond theoretical discussions, effectively bridging the gap between the theory and implementation of co‐creative processes. By focusing on inclusivity, we confront systemic barriers and champion the empowerment of minoritised students. This initiative exemplifies a robust co‐creation methodology that addresses the unique challenges faced by minoritised students and serves as a dynamic model for future educational partnerships. Despite its complexities, the co‐creation approach proves to be an effective means of advancing inclusivity and counteracting discriminatory practices within physiotherapy education.

## Author Contributions

**Yetunde M. Dairo:** conceptualization (equal), funding acquisition (equal), methodology (equal), formal analysis (equal), project administration (lead), resources (equal), writing – original draft (lead), writing – review and editing (lead). **Meriel Norris:** conceptualization (equal), funding acquisition (equal), methodology (equal), formal analysis (equal), project administration (co‐lead), resources (equal), writing – original draft (supporting), writing – review and editing (supporting). **Annabel Williams:** conceptualization (equal), funding acquisition (equal), methodology (equal), formal analysis (equal), resources (equal), writing – original draft (supporting), writing – review and editing (supporting). **John A. Hammond:** conceptualization (equal), funding acquisition (equal), methodology (equal), formal analysis (equal), project administration (co‐lead), resources (equal), writing – original draft (supporting), writing – review and editing (supporting).

## Ethics Statement

We ensured that the project respected participants' confidentiality, adhered to ethical guidelines, and contributed to the academic community with honesty and integrity.

## Conflicts of Interest

The authors declare no conflicts of interest.

## Supporting information

Supplementary_material_Clean.

## Data Availability

The data supporting this study are not publicly available due to confidentiality agreements with participants. However, anonymised data may be shared upon reasonable request to the corresponding author.

## References

[1] C. Bovill . 2020. Co‐Creating Learning and Teaching: Towards Relational Pedagogy in Higher Education.

[2] C. K. Prahalad and V. Ramaswamy , “Co‐Creation Experiences: The Next Practice in Value Creation,” Journal of Interactive Marketing 18, no. 3 (2004): 5–14, 10.1002/dir.20015.

[3] S. L. Vargo and R. F. Lusch , “Institutions and Axioms: An Extension and Update of Service‐Dominant Logic,” Journal of the Academy of Marketing Science 44, no. 1 (2016): 5–23, 10.1007/s11747-015-0456-3.

[4] A. Guachalla and M. Gledhill , “Co‐Creating Learning Experiences to Support Student Employability in Travel and Tourism,” Journal of Hospitality, Leisure, Sport & Tourism Education 25 (2019): 100210, 10.1016/j.jhlste.2019.100210.

[5] E. B. N. Sanders and P. J. Stappers , “Co‐Creation and the New Landscapes of Design,” CoDesign 4, no. 1 (2008): 5–18, 10.1080/15710880701875068.

[6] M. Steen , M. Manschot , and N. D. Koning , “Benefits of Co‐Design in Service Design Projects,” International Journal of Design 5, no. 2 (2011): 53–60.

[7] S. Apers , H. Vandebosch , T. Perko , and N. Železnik , “Co‐Designing Communication: A Design Thinking Approach Applied to Radon Health Communication,” International Journal of Environmental Research and Public Health 20, no. 6 (2023): 4965, 10.3390/ijerph20064965. 36981872 PMC10048842

[8] T. Morse , K. Luwe , K. Lungu , et al., “A Transdisciplinary Methodology for Introducing Solar Water Disinfection to Rural Communities in Malawi: Formative Research Findings,” Integrated Environmental Assessment and Management 16, no. 6 (2020): 871–884, 10.1002/ieam.4249. 32048797 PMC7687190

[9] R. Corcoran , G. Marshall , and E. Walsh , “The Psychological Benefits of Cooperative Place‐Making: A Mixed Methods Analyses of Co‐Design Workshops,” CoDesign 14, no. 4 (2018): 314–328, 10.1080/15710882.2017.1340484.

[10] W. Waheed , N. Mirza , M. W. Waheed , et al., “Recruitment and Methodological Issues in Conducting Dementia Research in British Ethnic Minorities: A Qualitative Systematic Review,” International Journal of Methods in Psychiatric Research 29, no. 1 (2020): e1806, 10.1002/mpr.1806. 31808215 PMC7051842

[11] Y. M. Dairo , M. Norris , J. A. Hammond , and A. Williams , “Developing a Training Resource for Racial Inclusivity in Physiotherapy Education: Findings From Pilot Work and Evaluation,” in *World Physiotherapy Congress Proceedings* (2023), https://world.physio/congress-proceeding/developing-training-resource-racial-inclusivity-physiotherapy-practice-0.

[12] C. Frazier , “It's More Than Just News: Print Media, the Tuskegee Syphilis Study and Collective Memory Among African Americans,” Journal of Historical Sociology 33, no. 3 (2020): 280–296, 10.1111/johs.12281.

[13] M. Hussain‐Gambles , K. Atkin , and B. Leese , “Why Ethnic Minority Groups Are Under‐Represented in Clinical Trials: A Review of the Literature,” Health and Social Care in the Community 12, no. 5 (2004): 382–388, 10.1111/j.1365-2524.2004.00507.x. 15373816

[14] A. Smart and E. Harrison , “The Under‐Representation of Minority Ethnic Groups in UK Medical Research,” Ethnicity & Health 22, no. 1 (2017): 65–82, 10.1080/13557858.2016.1182126. 27174778

[15] L. J. C. Ansley and R. Hall , “Freedom to Achieve: Addressing the Attainment Gap Through Student and Staff Co‐Creation,” Compass: Journal of Learning and Teaching 12, no. 1 (2019): 1–15, 10.21100/compass.v12i1.946.

[16] Chartered Society of Physiotherapy (CSP) . 2023. *Annual Quality Review 2021/2022: UK Pre‐Registration Physiotherapy Education*. Report: PD141, accessed April 10, 2024, https://www.csp.org.uk/system/files/publication_files/Annual%20Quality%20Review%2021-22_0.pdf.

[17] Office for National Statistics (OFS) . 2021. *Census 2021*, accessed April 10, 2024, https://www.ons.gov.uk.

[18] K. Bhopal , “Complicity and Conformity: Perpetuating Race and Class Hierarchies in UK Higher Education,” Educational Review (2024): 1–15, 10.1080/00131911.2024.2398754.

[19] M. Norris , J. A. Hammond , A. Williams , R. Grant , S. Naylor , and C. Rozario , “Individual Student Characteristics and Attainment in Pre‐Registration Physiotherapy: A Retrospective Multi‐Site Cohort Study,” Physiotherapy 104, no. 4 (2018): 446–452, 10.1016/j.physio.2017.10.006. 29352580

[20] J. A. Hammond , A. Williams , S. Walker , and M. Norris , “Working Hard to Belong: A Qualitative Study Exploring Students From Black, Asian and Minority Ethnic Backgrounds' Experiences of Pre‐Registration Physiotherapy Education,” BMC Medical Education 19, no. 372 (2019): 372, 10.1186/s12909-019-1821-6. 31619242 PMC6794793

[21] J. A. Hammond , M. Norris , A. Williams , and Y. M. Dairo , Racial Inclusivity in Practice Education Training Resource (NHS Learning Hub, 2023), https://learninghub.nhs.uk/Resource/35702/Item.

[22] Y. M. Dairo , J. A. Hammond , D. Ishani , M. Norris , and A. Williams . 2022. A Racial Inclusivity Training Resource for Physiotherapy Practice Education: Evaluation Report 2022, https://www.hee.nhs.uk/sites/default/files/documents/HEE%20racial%20inclusivity%20practice%20education%20Evaluation%20Report%202022%20.pdf.

[23] A. A. Knapp , A. J. Carroll , N. Mohanty , et al., “A Stakeholder‐Driven Method for Selecting Implementation Strategies: A Case Example of Pediatric Hypertension Clinical Practice Guideline Implementation,” Implementation Science Communications 3, no. 1 (2022): 25, 10.1186/s43058-022-00276-4. 35256017 PMC8900435

[24] A. Hutchinson and A. Lovell , “Participatory Action Research: Moving Beyond the Mental Health ‘Service User’ Identity,” Journal of Psychiatric and Mental Health Nursing 20, no. 7 (2013): 641–649, 10.1111/jpm.12001. 23167824

[25] A. McIntyre , Participatory Action Research (Sage, 2008).

[26] T. Brown , “Design Thinking,” Harvard Business Review 86, no. 6 (2008): 84–92. 18605031

[27] U. Müssener , P. Henriksson , C. Gustavsson , et al., “Promoting Healthy Behaviors Among Adolescents and Young Adults With Intellectual Disability: Protocol for Developing a Digital Intervention With Co‐Design Workshops,” JMIR Research Protocols 12 (2023): e47877, 10.2196/47877. 37505807 PMC10422167

[28] A. V. Norström , C. Cvitanovic , M. F. Löf , et al., “Principles for Knowledge Co‐Production in Sustainability Research,” Nature Sustainability 3 (2020): 182–190, 10.1038/s41893-019-0448-2.

[29] Institute for Translational Health Sciences (ITHS) . 2021. Best Practices for Equitable Research at Each Step of the Research Process, accessed February 29, 2024, https://www.iths.org.

[30] National Health Service (NHS) England . 2023. *Co‐Production and Quality Improvement—A Resource Guide*. Ref PAR1498_iv, accessed May 24, 2024, https://www.england.nhs.uk/long-read/co-production-and-quality-improvement-a-resource-guide/.

[31] Kelley, T. , and D. Kelley , Creative Confidence: Unleashing the Creative Potential Within us all (Crown Currency, 2013).

[32] C. Y. Huang , L. Y. Tsai , C. H. Chung , F. F. Shih , and Y. M. Wang , “The Effect of Design Thinking Approach in Interprofessional Education Programme of Human Sexuality Course: A Quasi‐Experimental Design,” Nursing Open 10, no. 2 (2023): 967–976, 10.1002/nop2.1363. 36114695 PMC9834516

[33] S. R. Velu , “Design Thinking Approach for Increasing Innovative Action in Universities: ICT's Mediating Effect,” Sustainability 15, no. 1 (2023): 24, 10.3390/su15010024.

[34] Norris, M. , J. A. Hammond , A. Williams , and S. Walker , “Student Explorations of Black and Minority Ethnic Attainment Inequalities in Pre‐Registration Physiotherapy: A Qualitative Study,” supplement, Physiotherapy 105, no. S1 (2019): e1–e53.

[35] O. Ehie , I. Muse , L. Hill , and A. Bastien , “Professionalism: Microaggression in the Healthcare Setting,” Current Opinion in Anaesthesiology 34, no. 2 (2021): 131–136, 10.1097/ACO.0000000000000966. 33630771 PMC7984763

[36] C. Davis and L. Parmenter , “Student‐Staff Partnerships at Work: Epistemic Confidence, Research‐Engaged Teaching, and Vocational Learning in the Transition to Higher Education,” Educational Action Research 29, no. 2 (2020): 292–309, 10.1080/09650792.2020.1792958.

[37] A. de Bie , E. Marquis , A. Cook‐Sather , and L. Luqueño , “Valuing Knowledge(s) and Cultivating Confidence: Contributions of Student‐Faculty Pedagogical Partnerships to Epistemic Justice,” Innovations in Higher Education Teaching and Learning 16 (2019): 35–48, 10.1108/S2055-364120190000016004.

[38] T. M. Lubicz‐Nawrocka , “Students as Partners in Learning and Teaching: The Benefits of Co‐Creation of the Curriculum,” International Journal for Students as Partners 2, no. 1 (2018): 47–63, 10.15173/ijsap.v2i1.3207.

[39] A. Kehler , R. Verwood , and H. Smith , “We Are the Process: Reflections on the Underestimation of Power in Students as Partners in Practice,” International Journal for Students as Partners 1, no. 1 (2017): 38–52, 10.15173/ijsap.v1i1.3176.

[40] J. Owen and C. Wasiuk , “An Agile Approach to Co‐Creation of the Curriculum,” International Journal for Students as Partners 5, no. 2 (2021): 89–97, 10.15173/ijsap.v5i2.4475.

[41] J. M. Chevalier and D. J. Buckles , Participatory Action Research. Theory and Methods for Engaged Enquiry (Taylor and Francis, 2013).

[42] A. B. Bjørnerås , E. Langørgen , A. E. Witsø , L. Kvam , A. E. Leithaug , and S. Horghagen , “Aiming for Inclusion: Processes Taking Place in Co‐Creation Involving Students With Disabilities in Higher Education,” International Journal of Inclusive Education 28, no. 14 (2023): 3437–3453, 10.1080/13603116.2023.2230198.

[43] T. M. Lubicz‐Nawrocka , “‘More Than Just a Student’: How Co‐Creation of the Curriculum Fosters Third Spaces in Ways of Working, Identity, and Impact,” International Journal for Students as Partners 3, no. 1 (2019): 34–49.

[44] S. Shakir and A. Siddiquee , “‘Our Community Building and Belonging’: A Student and Staff Co‐Creation Project,” Equity in Education & Society (2023): 4–17, 10.1177/27526461231166013.

