# Corection to “Co‐Creation Methodology for Developing a Racial Inclusivity Training Resource in Physiotherapy Education”

**DOI:** 10.1111/hex.70473

**Published:** 2025-10-31

Y. Dairo, M. Norris, A. Williams, and J. A. Hammond, “Co‐Creation Methodology for Developing a Racial Inclusivity Training Resource in Physiotherapy Education”, *Health Expectations: An International Journal of Public Participation in Health Care and Health Policy* 28, no. 4 (2025): e70363.

In the article cited above, John A. Hammond's affiliation was published as “Centre for Allied Health, St George's University of London, London, UK”.

It should be:

School of Allied Health Professions, Public Health and Social Work, Canterbury Christ Church University, Canterbury, UK

Additionally, John A. Hammond's ORCID ID is: https://orcid.org/0000-0001-5246-426X

We apologize for this error.

