# Supplementary material for: Co‐Creation Methodology for Developing a Racial Inclusivity Training Resource in Physiotherapy Education
Source: Health Expect. 2025 Aug 4;28(4):e70363. doi: 10.1111/hex.70363 (PMC12319567; doi:10.1111/hex.70363)
Supplement: Supplementary file 1 — Supplementary_material_Clean. [file HEX-28-e70363-s001.docx]

Supplementary material

An outline of the Co-creators’ advertisement key requirements

*Position: Seeking physiotherapy students and practice educator co-creators.*

*Location: Buckinghamshire New University, High Wycombe, School of Health Care and Social Work.*

*Salary Range: £48.50 hourly rate for physiotherapist practice educators, £20.68 for students.*

*Contract Type: Fixed-term.*

*Number of Co-creators: 5 Physiotherapists and 5 students.*

*Closing Date for Application: 18-Oct-2021.*

*Project Description: Developing a new learning resource for supporting racial inclusivity in physiotherapy practice education.*

*Eligibility Criteria: Qualified physiotherapists in the UK with practice education experience or pre-registration physiotherapy students from a Black, Asian, and minority ethnic background who have completed at least one practice placement in the UK.*

*Expectations: Contribution to steering group activities, attending seven meetings over the project duration, preparation, co-designing resource content based on previous data, development, implementation, and evaluation.*

*Meetings: Seven 1-hour online meetings, tentatively scheduled for the 1st and 3rd Tuesday of each month between 5 and 6pm.*

*Enquiries and Application: Contact Dr. Yetunde Dairo at yetunde.dairo@bucks.ac.uk. Interested individuals should send a brief email outlining their intention, how they meet the criteria, and an up-to-date CV (not more than four pages for educators, two pages for students). Informal enquiries are welcome.*
